# P-1214. In Vitro Antibacterial Spectrum and Activity of Tebipenem Against Enterobacterales Clinical Isolates Causing Urinary Tract and Bloodstream Infections in the United States and United Kingdom in 2023-2024

**DOI:** 10.1093/ofid/ofaf695.1407

**Published:** 2026-01-11

**Authors:** Renuka Kapoor, Timothy Doyle, Zachary Kockler, Rodrigo mendes, Mariana Castanheira, Didem Torumkuney, Ian A Critchley

**Affiliations:** GSK, Atlanta, Georgia; Element Materials Technology/Jones Microbiology Institute, North Liberty, IA; Element Iowa City (JMI Laboratories), North Liberty, Iowa; Element Iowa City, North Liberty, Iowa; Element, North Liberty, IA; GSK, Atlanta, Georgia; Spero Therapeutics, Cambridge, Massachusetts

## Abstract

**Background:**

Tebipenem pivoxil hydrobromide (formerly SPR994) is in clinical development as potentially the first oral broad-spectrum carbapenem agent in the US for the treatment of complicated urinary tract infections (cUTI) and acute pyelonephritis (AP). This study reports on the *in vitro* activity of tebipenem and comparator agents tested against Enterobacterales isolates recovered from UTI and bloodstream infections (BSI) in the US.

**Figure:**
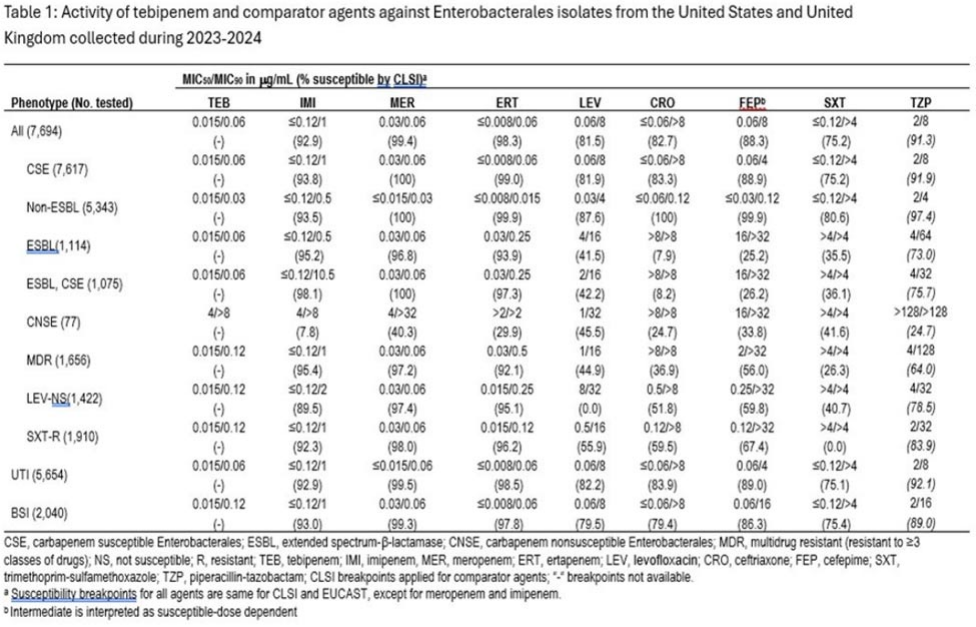

**Methods:**

Between 2023 and 2024, 7,694 Enterobacterales clinical isolates were collected from 72 medical centers in the US and UK. These included 73.5% (5,654) from UTI (54.8% (3101) outpatients; 28.7% (1624) inpatients, including 11% (179) hospital-acquired infections) and 26.5% (2,040) from BSI (22.4% (456) outpatients; 67% (1366) inpatients, including 19% (261) hospital-acquired infections). Isolates were tested for susceptibility by CLSI reference broth microdilution method. MIC results for comparator agents were interpreted using the most recent CLSI M100 (2025) breakpoint criteria.

**Results:**

Tebipenem MIC_50_ and MIC_90_ values against all 7,694 Enterobacterales isolates were 0.015 μg/mL and 0.06 μg/mL, respectively (UTI and BSI specific results are shown in the Table). Similar MIC_50/90_ values were obtained for ertapenem (MIC_50/90_, ≤0.008/0.06 μg/mL), imipenem (MIC_50/90_, ≤0.12/1 μg/mL), and meropenem (MIC_50/90_, 0.03/0.06 μg/mL). The susceptibility rates for recommended comparator agents were below 89% for levofloxacin (81.5%, MIC_50/90_, 0.06/8 μg/mL), ceftriaxone (82.7%, MIC_50/90_, ≤0.06/ >8 μg/mL), cefepime (88.3%, MIC_50/90_, 0.06/8 μg/mL, and trimethoprim-sulfamethoxazole (75.2%, MIC_50/90_, ≤0.12/ >4 μg/mL). Tebipenem remained active against isolates resistant to other agents, including ESBL or MDR phenotypes, with MIC_50_ and MIC_90_ values of 0.015 µg/mL and 0.06-0.12 µg/mL, respectively. Antibacterial agents from other drug classes showed susceptibility rates of between 0% and 67%.

**Conclusion:**

Tebipenem had potent *in vitro* activity against a diverse set of Enterobacterales clinical isolates from the US, including those with ESBL and MDR phenotypes. These results indicate that tebipenem has activity comparable to IV carbapenems and can be a potential oral agent for treatment of cUTI and AP.

**Disclosures:**

Renuka Kapoor, PhD, GSK: Employee|GSK: Stocks/Bonds (Public Company) Mariana Castanheira, PhD, Melinta Therapeutics: Advisor/Consultant|Melinta Therapeutics: Grant/Research Support Didem Torumkuney, PhD, GSK: Stocks/Bonds (Public Company) Ian A. Critchley, PhD, Spero Therapeutics: Stocks/Bonds (Public Company)

